# “Real-Time” Monitoring of Under-Five Mortality: Lessons for Strengthened Vital Statistics Systems

**DOI:** 10.1371/journal.pmed.1001904

**Published:** 2016-01-25

**Authors:** Jennifer Bryce, Agbessi Amouzou, Cesar G. Victora, Gareth Jones, Romesh Silva, Kenneth Hill, Robert E. Black, RMM Working Group

**Affiliations:** 1 Institute for International Programs, Johns Hopkins Bloomberg School of Public Health, Baltimore, Maryland, United States of America; 2 Data & Analytics Section, UNICEF, New York, New York, United States of America; 3 University of Pelotas, Pelotas, Brazil; 4 Adeni Consulting Inc., Ottawa, Canada

## Abstract

Bryce and colleagues, reflect on lessons that can be learned from the Real-Time Monitoring of Under-Five Mortality Collection.

**Summary Points**

- Calls for improved civil registration and vital statistics systems as a central part of the development agenda for low-income countries have noted the absence of evidence on the feasibility and accuracy of such systems.
- We synthesize findings from a seven-year project in Ethiopia, Ghana, Malawi, Mali, and Niger that tested methods for the real-time monitoring of under-five mortality (RMM), with particular attention to their implications for strengthening vital statistics systems.
- We tested three broad approaches, and found that none offers a surefire approach to obtaining accurate information on child deaths for recent periods of one year or less.
- Those working to strengthen vital statistics systems should anticipate three ongoing challenges: ensuring data quality; addressing local challenges to community-based reporting; and planning for open data access.
- This Collection underscores the importance of comprehensive reporting of results, including negative results. Full documentation and reporting of efforts to improve vital statistics systems are needed to inform future efforts.

## Introduction

Civil registration and vital statistics systems are increasingly recognized as drivers of human rights, health, and development programs—especially for women and children [1]. Vital statistics systems are weak in most low-income countries [2,3], precisely where the burden of maternal and child deaths is highest and, therefore, the need for up-to-date, high-quality estimates of levels and trends in maternal and child mortality is greatest [4]. The UN Commission on Information and Accountability for Women’s and Children’s Health identified the reporting of births and deaths as a cornerstone for vital statistics systems in low-income countries [5]. This has led to intensified efforts to support and strengthen the routine reporting of vital events [6].

In this paper, we synthesize results generated by the Real-Time Monitoring of Under-Five Mortality (RMM) project, and highlight aspects that can inform efforts to strengthen vital statistics systems in low-income countries. Table 1 provides an overview of the methods tested by the RMM project in Ethiopia, Ghana, Malawi, Mali, and Niger. Full details on the design and implementation of RMM methods, the accuracy of the mortality estimates they produce, and their running costs are reported in the papers that form the Collection.

**Table 1:** Real-time mortality monitoring (RMM) approaches tested, by country.

	Community-based reporting on vital events		Adjusted health facility data	Rapid household survey methods	
	*Paid worker*	*Unpaid worker*		*Repeated*	*Imputed*
**Ethiopia**	✓			✓	✓
**Ghana**		✓			✓
**Malawi**	✓		✓	✓	✓
**Mali**		✓			✓
**Niger**				✓	✓

The RMM project produced two sets of overarching findings: 1) quantitative results on the accuracy of under-five mortality estimates produced by various methodological approaches; and 2) experiential findings on the challenges of generating vital statistics for recent periods in real-world settings.

## Quantitative Findings

We tested three broad approaches in 13 discrete applications conducted across five country settings [7]. We assessed accuracy of mortality measures by comparison to household survey data from full birth histories, described in this paper as current best practice. Only three of these applications—reporting of vital events by community-based workers (CBWs) in Mali [8], and birth history imputation methods in Niger and Malawi [9]–produced estimates of under-five mortality rates for recent one-year periods that we consider sufficiently accurate and stable to support sound public health decision-making.

We tested community-based methods for the reporting of vital events in four countries (Ethiopia, Ghana, Malawi, and Mali) [10]. The results from Ghana are still under review due to data quality concerns; results from the other countries are reported in the papers that comprise the Collection. In Ethiopia and Malawi, under-five deaths reported by CBWs represented only 22% and 51%, respectively, of those identified by current best practice survey data [10]. Even more troubling is that in Malawi, where we were able to analyze multiple 12-month periods, the levels of underestimation were not stable, vitiating the potential for using standardized adjustments to produce estimates of trends that are good enough to support decision-making [11,12].

In Mali, the CBW method produced highly accurate estimates of mortality, essentially identical to those produced by the best practice census, although both births and deaths were slightly underestimated (e.g., 91% of the under-five deaths identified by a best practice census). Why did the CBW-based method succeed in Mali and not elsewhere? At the request of the Ministry of Health and our local research collaborators, we conducted the testing of the CBW-based method in Mali as more of a rigorous trial in a smaller geographic area than in the other three countries, which means that the results are likely to be more difficult to replicate at scale [8]. The running cost per 1,000 population for the CBW method in Mali ($9,141) was five times higher than in Malawi ($1,736) and 14 times higher than in Ethiopia ($645) [9], and was supported by the use of financial incentives to motivate the CBWs, frequent supervision, and data quality controls at every level [8].

We tested a second RMM method that used child deaths recorded in health facilities and adjusted them based on data about where a child died, collected through a household survey. We tested this only in Malawi, because it was the only RMM setting in which more than 50% of births were reported as occurring in facilities at the time the study was designed [13]. The results were disappointing, and were published in advance of this Collection [11]. We found that under-five mortality rates generated by adjusting health facility data for reported proportions of births and child deaths in facilities were between 35% and 65% of those estimated by the current best practice survey in one RMM district (Balaka), and between 46% and 50% in the other RMM district (Salima), for four overlapping 12-month periods in 2010–2011. Our conclusion is that in Malawi, despite a high level of institutional births relative to other low-income sub-Saharan countries, routine data on births and deaths cannot be used at present to estimate annual trends in under-five mortality. Others point to the potential of using health service platforms to strengthen CRVS systems and generate real-time information [14]. Our experience suggests that considerable time and effort will be needed to assess and address the completeness and quality of facility-based data before they can be used as a reliable source of vital statistics.

Finally, we tested two different approaches based on summary birth histories in household surveys. These methods are attractive because they would allow countries to make use of existing data sources with minimal additional investment, and would be less expensive than mounting a full birth history survey. One method uses changes in children ever born and children dead between successive surveys to produce mortality estimates for the inter-survey period. The second method imputes full birth histories from an earlier survey onto the summary birth histories from a current survey, and then analyzes the imputed full birth histories using standard methods; this method is essentially an improvement on existing indirect methods, but cannot produce true period-specific estimates. Unfortunately, our findings suggest that neither method holds promise for generating reliable real-time estimates. The first method was particularly disappointing, producing nonsensical results (negative changes in numbers of children dead) in five out of six applications. The second method, which in principle produces estimates for each of the 10 calendar years before the second survey, and thus gives a sense of time trend from a single application, worked well in two applications (Niger, with a mean relative error for the 10 estimates vis-a-vis the estimates produced by the current best practice survey of zero, and zero estimates outside the range of ± 20%; and Malawi, with comparable figures of 0.02 mean relative error and one estimate outside the ± 20% range). Other applications were less successful. The worst example was Mali (ironically, given the country’s success with CBWs), with a mean relative error of -0.21 and five out of 10 annual estimates at least 20% below the current best practice value. The most important limiting factor in both methods is the underlying quality of the summary birth histories. With high-quality summary histories, the imputation method gives plausible results, even for trends, although strictly speaking they are not true period estimates; the inter-survey change method, however, is just too sensitive to data quality to give stable estimates.

The quantitative results therefore suggest that none of these methods—selected by global experts as having the most promise, and confirmed as likely to be feasible by counterparts in participating countries—can be unequivocally endorsed as the “way to go” to ensure that governments and their partners have the evidence they need to track short-term changes in child survival. Applying a common metric of accuracy (annualized under-five risks of dying produced by the method as a proportion of those produced by a best practice household survey or census), the results for CBW-based approaches range from a high of 91% in Mali to a low of 22% in Ethiopia; the results for the single Malawi assessment of the facility-based method produced a mean accuracy of 50% across the two participating districts. Across the six applications of the two survey methods, five produced logically impossible results (although for the exception, Malawi, the estimate was 94% of the best practice estimate); and for the six applications of the imputation approach, which in total produced 60 annual estimates, 44 were within ± 20%, 9 were too low (< 80%) and 7 were too high (> 120%).

## Challenges Encountered in Implementing the RMM Project

The RMM project has produced some of the only evidence drawn from systematic experience with implementing and evaluating alternative methods for tracking births and deaths. The results must be generalized with care, because RMM sites were selected based on plans for child survival programming in Africa, and may not hold true in other regions. Still, our aim was to put our proverbial scientific shoulder against the challenge of real-time monitoring of mortality, and we offer the following experiential lessons for those who are working to strengthen vital statistics systems.

One lesson, stated often in the past but worth repeating here, is that data quality is paramount. We found that even the current best practice method of collecting full birth histories through large-scale household surveys often produces results that do not meet demographic standards for data quality. The field teams working on the CBW reporting methods in Ethiopia, Malawi, and Mali worked hard to identify and address data quality issues. As described in the country-specific papers [8,15,16], one of the most challenging data quality issues in Mali was ensuring that the CBWs followed up every pregnancy to determine its outcome. In Malawi, challenges included frequent turnover of RMM-trained CBWs, requiring frequent refresher training, and broader health system failures that led to stockouts in reporting forms and the airtime needed to transmit reports. Steps taken by the RMM study teams to address these shortcomings included regular supervision visits, rechecks of samples of recorded events by visiting households, and the introduction of various incentive schemes.

The RMM experience also highlights the need for caution in implementing vital statistics systems that may look good on paper, but do not work in the real world. In each of the five country settings, there were specific challenges that required modifications in the RMM design and analysis plans. For example, one challenge that manifested itself in different ways across multiple sites was low literacy and particularly numeracy skills, not only among CBWs but also among clinicians participating in the test of the health facility method in Malawi. RMM teams found creative ways to address these deficits, ranging from assigning a second, literate “companion” to the CBW in Ghana to the use of specially-designed recording forms in Mali. Another example of a common challenge that appeared differently across sites was ensuring that the RMM-trained CBW was present in and trusted by the community. Staff turnover, poor recruitment procedures, ineffective incentives, and competing priorities in the CBW workload all posed problems early in RMM implementation that individual sites needed to address. Those planning for strengthened vital statistics systems will need to include a formative period with close monitoring in order to ensure that those recording vital events have the capacity to do so correctly.

A third and somewhat different challenge, this time at project level, was ensuring open data access. Recent and welcome requirements by journals that datasets be available to support replication and secondary analyses proved more time- and resource-intensive to implement than anticipated, although the actual financial cost charged by the Johns Hopkins Data Management Services was only US$ 1600 for five years. In terms of human resources, however, we invested considerable time and expertise to design an appropriate data system architecture, especially because open access was not taken into account in designing RMM data collection protocols, and the data points are drawn from disparate sources. Country capacity for data management in our partner institutions was limited, and required special training and close supervision, especially in de-identifying the data in preparation for archiving. A particular challenge for RMM was that in most countries, the project was carried out in a small number of districts, and eliminating standard identifying information was not sufficient for anonymization. In the future, aligning in-country and technical partner plans with donor and journal requirements for open data access should be an integral part of all project proposals.

## The Way Forward

CRVS systems have many worthy objectives, some of which are at the level of the individual, such as human rights, and some of which are at population level, such as vital statistics. For population level objectives, the data must be representative, complete, and accurate.

Most CVRS systems are passive, requiring individuals to come to a government office to register a birth or death. The modest successes with birth registration are attributable, at least in part, to the fact that it was tied in most of these settings to concrete benefits for families. Death registration does not confer these advantages. The RMM CBW approaches used active seeking of birth and child death events, which may be a useful complement to more comprehensive CRVS systems aiming to generate valid statistics on fertility and mortality. Although our results are mixed, more experience is needed with linking reporting of vital events by CBWs with a formal system of civil registration. One way forward would be for countries to begin vital event enumeration in a representative sample of areas, where attention to completeness and quality of reporting may produce usable statistics in the short term. In addition, these initial experiences can generate valuable new knowledge about how various approaches to data collection, including the use of CBWs, can lead to systems able to be implemented at scale.

An important dimension of child mortality not addressed explicitly by the RMM project is cause of death. Monitoring aggregate child mortality trends is important, but the information becomes of much greater programmatic value if deaths can be disaggregated by cause. Although simply counting births and deaths is fundamental to understanding the needs of communities, much more valuable would be obtaining information on the likely medical causes and social and health system contributors to death. Medical certification of the underlying cause of death is part of a complete CRVS system, but will be difficult to accomplish uniformly in the near future because in low-income countries, up to 80% of deaths occur outside of health facilities. An alternative method for determining the medical cause of death is a post-mortem interview—a so-called verbal autopsy—that can be administered by lay workers to family members of the deceased. A further addition to asking about the signs and symptoms of the fatal illness is asking about the social conditions of the household, illness recognition, care seeking, and experience with accessing the health system or alternative providers for the illness. This has been shown to provide valuable findings on the need to reach underserved populations, improve knowledge about illness and danger signs, enhance appropriate seeking of care, and improve health system access and quality of care and follow-up. Methods for monitoring child mortality need to be judged by their potential for providing a platform for verbal and social autopsies, as well as by their accuracy. The CBW approach clearly provides this opportunity, but the health facility approach and the survey methods do not, because they do not identify individual deaths.

We need to work toward complete civil registration and vital statistics systems in low-income countries, using innovative approaches [14] after rigorous evaluation, and recognizing that long-term efforts will be required. More research can help define the mechanisms needed to improve and build confidence in the completeness and quality of both facility and community-based reporting of mortality. Generating a comprehensive evidence base that can serve as a repository for negative as well as positive results is an essential part of that work. RMM provides a useful example of the need for comprehensive reporting, including negative results [17]. We hope that this Collection can serve as a platform for collaboration and continuing research in this area.
